# Negative Pressure Ventilation via Biphasic Cuirass Device in an Adolescent With Congenital Central Hypoventilation Syndrome Undergoing Septorhinoplasty: A Case Report

**DOI:** 10.7759/cureus.112606

**Published:** 2026-07-13

**Authors:** Rohan S Bellary, Michael Orangias, Ronald Morton, Sarah Khayat, Egambaram Senthilvel

**Affiliations:** 1 Sleep Medicine and Otolaryngology, University of Louisville, Louisville, USA; 2 Pulmonology, University of Louisville, Louisville, USA; 3 Pediatrics, University of Louisville, Louisville, USA; 4 Facial Plastics, University of Louisville, Louisville, USA

**Keywords:** biphasic cuirass ventilation, congenital central hypoventilation syndrome, negative pressure ventilation, septorhinoplasty, sinus pause

## Abstract

Congenital central hypoventilation syndrome (CCHS) is characterized by absent ventilatory responses to hypercapnia and hypoxemia due to paired-like homeobox 2B (PHOX2B) mutations, requiring lifelong nocturnal ventilatory support. Out of the four current management methods, biphasic cuirass ventilation (BCV) is the only one that avoids the use of a face-mounted interface. We present an 18-year-old man with PHOX2B-confirmed CCHS who required perioperative bridging from home average volume-assured pressure support (AVAPS) to BCV following septorhinoplasty with rib cartilage grafting, which precluded mask usage for 6-8 weeks. BCV was used for two months during the perioperative period, and home AVAPS was eventually resumed. The case was complicated by perioperative sinus pauses requiring leadless pacemaker implantation. This case demonstrates that BCV provides effective nocturnal ventilatory support in CCHS in settings when mask interfaces are contraindicated and highlights the need for postoperative cardiac monitoring in PHOX2B mutation carriers.

## Introduction

Congenital central hypoventilation syndrome (CCHS) is a rare disorder of autonomic respiratory control, with an estimated incidence of 1 in 150,000 to 200,000 live births and a global prevalence of at least 3,000 cases [1]. The condition results from mutations in the paired-like homeobox 2B (PHOX2B) gene, a transcription factor that plays a role in differentiation and maintenance of brainstem neurons in the retrotrapezoid nucleus (RTN), neurons essential for chemoreception and ventilatory responses to hypercapnia and hypoxemia [2,3]. Loss of RTN function impairs CO₂ detection during sleep, driving recurrent nocturnal hypoventilation that can progress to cardiorespiratory failure. CCHS is broadly understood as a disorder of neural crest cell development.

Current management of CCHS includes four ventilatory modalities: tracheostomy-based positive pressure ventilation, non-invasive positive pressure ventilation (NIV) via mask, phrenic nerve pacing, and negative pressure ventilation (NPV) [4]. Negative pressure devices such as the biphasic cuirass ventilator (BCV) are infrequently used in CCHS, with minimal study. No standardized protocol exists for perioperative ventilation in CCHS patients whose nasal anatomy or surgical grafting inhibits mask use. We present a patient who successfully bridged the postoperative period following septorhinoplasty using BCV, with a notable additional finding of perioperative sinus pauses requiring pacemaker implantation.

## Case presentation

The patient is an 18-year-old white, non-Hispanic male with CCHS associated with a confirmed heterozygous PHOX2B mutation. He underwent tracheostomy at three months of age and was subsequently transitioned to nocturnal NIV using average volume-assured pressure support (AVAPS) (expiratory positive airway pressure (EPAP) 6 cm H₂O, inspiratory positive airway pressure (IPAP) 10-20 cm H₂O, backup rate 16 breaths/min, and tidal volume 781 mL) after tracheostomy decannulation at the age of nine years. PHOX2B testing was performed twice: equivocal at three years and confirmatory at 11 years. He has been primarily managed by sleep medicine and pulmonary teams. He was followed by oncology for neural crest tumor risk, with no history of tumors developed thus far. He was also seen by cardiology and had an echocardiogram in 2021. Past surgical history included adenotonsillectomy, Nissen fundoplication, tympanostomy tube placement, and gastrostomy with subsequent removal. At five years of age, he had an accidental bike injury, which caused nasal deformity at the time without significant impairment. However, through adolescence, he gradually started to have significant nasal obstruction and functional difficulty because of worsening septal deviation and further external deformity. At the age of 15 years, his symptoms worsened, causing concern for both functional and cosmetic disparities.

At the age of 16 years, he was initially evaluated by otolaryngology (ENT). ENT examination findings noted a prominent bony dorsal hump, upper and middle third nasal deviation to the left, tip ptosis, and severe caudal septal deviation in a C-shaped configuration. The Cottle maneuver was positive on the left. Nasal endoscopy showed approximately 85% obstruction on the left with no polyps or purulence. He was considered a candidate for functional open septorhinoplasty with Musculoskeletal Transplant Foundation (MTF) cadaveric rib cartilage grafting when he turned 18 years of age.

Because cartilage grafts would prevent mask-based ventilation for 6-8 weeks postoperatively, sleep medicine and pulmonary medicine discussed options for different interfaces. Shared decision-making resulted in the decision to use negative thoracic ventilation as a temporary measure. Alternative strategies were considered before selecting BCV. Temporary tracheostomy was felt to be unnecessarily invasive given the patient's stable long-term reliance on nocturnal NIV alone and the temporary nature of the interface conflict. Similarly, phrenic nerve pacing was not pursued, as it required surgical implantation and is intended for long-term ventilatory dependence rather than a temporary perioperative need. Alternative NIV mask interfaces were considered but were not feasible options, as postoperative nasal splinting and cartilage grafting precluded adequate mask fit and risked surgical repair compromise in the critical perioperative recovery period. BCV was therefore selected as the least invasive option capable of providing adequate nocturnal ventilatory support while preserving the surgical outcome.

The patient trialed this device at an outpatient setting 10 months prior to his surgery and decided to move forward once insurance approval was obtained after several appeals. He had an initial overnight titration study with BCV to determine an optimal regimen (Table 1). Hypoventilation was stabilized at the setting of −22/+8 cm H₂O, backup rate 16 breaths/min, 1:1 synchrony, and trigger 8. He further acclimated with this device for two weeks prior to the surgery. Preoperative arterial blood gas (ABG) on home AVAPS showed pH 7.395, pCO₂ 46.2 mmHg, pO₂ 94.6 mmHg, HCO₃⁻ 28.3 mmol/L, and O₂ saturation 98.2%. Given the severity of his condition, postoperative intensive-care unit (ICU) monitoring was arranged.

**Table 1:** Serial polysomnography results. AHI: apnea-hypopnea index; NREM: non-rapid eye movement; REM: rapid eye movement; TST: total sleep time; BCV: biphasic cuirass ventilator; AVAPS: average volume-assured pressure support; NPSG: nocturnal polysomnography.

Parameter	Baseline NPSG (age ten-year old)	Pre-op BCV titration (02/13/24)	Post-op AVAPS titration (05/08/25)	Normal
AHI (events/hour)	11.3	0.8	1.0	<1.5
SpO₂ nadir (%)	83	95	82	≥90
Mean SpO₂ (%)	93	98	98	≥95
Peak CO₂ (mmHg)	71	60	51	≤50
Mean CO₂ NREM (mmHg)	63	55	44	≤45
Mean CO₂ REM (mmHg)	--	55	41	≤45
TST with CO₂ >50 mmHg (%)	100%	100%	5.76%	<25%

**Figure 1:**
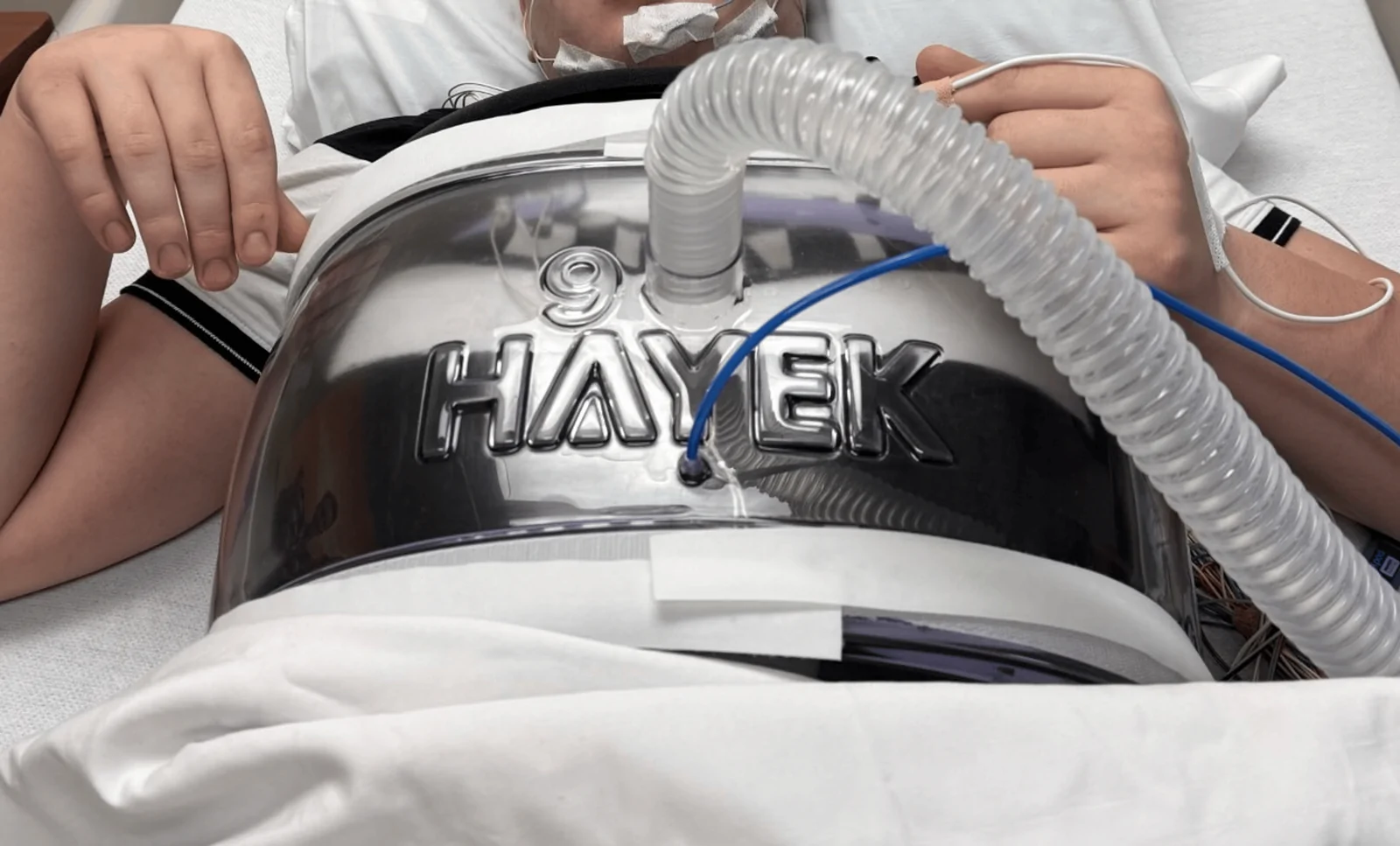
Patient wearing biphasic cuirass ventilation (BCV) negative thoracic pressure ventilation device postoperativley. Patient utilizing the Hayek RTX biphasic cuirass ventilator (Hayek Medical Devices, Philadelphia, Pennsylvania) during the postoperative period. As shown, the rigid cuirass shell positioned over the anterior thorax generates alternating negative and positive extrathoracic pressures to facilitate inspiration and expiration without the need for a facial or nasal interface [6], making it suitable after nasal reconstructive surgery that precludes mask usage.

Septorhinoplasty was performed in February 2025 without intraoperative complications. During the procedure, 2 L of intravenous fluids were administered, with 2 L of urine output recorded. Intraoperatively, 30 mg of ketamine with a remifentanyl IV drip and 50 mg of fentanyl were administered with noted hemodynamic stability and no anesthetic complications. Postoperative course and ventilator settings are summarized in Table 2, with device placement in the ICU (Figure 1). On postoperative day (POD) 1, the patient experienced sinus slowing during a vomiting episode. An ABG obtained that day showed pH 7.31, pCO₂ 66 mmHg, pO₂ 90 mmHg, and HCO₃⁻ 34 mmol/L, consistent with acute-on-chronic hypoventilation. A 17-second sinus pause was recorded on telemetry on POD 2 during emesis, and an 11-second pause occurred on POD 3 while the patient was ambulating. Cardiology was consulted on POD 3 due to a known association between PHOX2B mutations and sinus pauses with risk of sudden cardiac death [5]. Following multidisciplinary discussion, a leadless pacemaker was placed on POD 6 without complications.

**Table 2:** Perioperative biphasic cuirass ventilation (BCV) settings and supplemental oxygen requirements. BCV: biphasic cuirass ventilator; AVAPS: average volume-assured pressure support; EPAP: expiratory positive airway pressure; IPAP: inspiratory positive airway pressure; V_T_: tidal volume; RR: respiratory rate; NIV: Non-invasive ventilation; FiO₂: fraction of inspired oxygen; POD: postoperative day; PSG: polysomnogram.

Day	BCV settings	Supplemental O₂ (Night)	Key events
Pre-op	AVAPS: IPAP 10-20 cm H₂O, EPAP 6 cm H₂O, V_T_ 781 mL, RR 16	None	Baseline NIV via nasal mask
POD 1	Rate 20 breaths/min, IPAP −24 cm H_2_O, EPAP +8 cm H_2_O	Simple mask 7 L/min	Poor adherence; sinus slowing with emesis; pCO₂ 66 mmHg
POD 2	Rate 20 breaths/min, IPAP −24 cm H_2_O, EPAP +8 cm H_2_O	Simple mask 7 L/min	17-second sinus pause on telemetry
POD 3	Rate 20 breaths/min, IPAP −24 cm H_2_​​​​​​​O, EPAP +8 cm H_2_​​​​​​​O	Simple mask 7 L/min	11-second pause while ambulating; cardiology consulted
POD 4	Rate 20 breaths/min, IPAP −24 cm H_2_​​​​​​​O, EPAP +8 cm H_2_​​​​​​​O	Simple mask 5 L/min	O₂ weaned
POD 5	Rate 20 breaths/min, IPAP −24 cm H_2_​​​​​​​O, EPAP +8 cm H_2_​​​​​​​O	Face tent 10 L/min, FiO₂ 50%	Transition to face tent
POD 6	Rate 20 breaths/min, IPAP −24 cm H_2_​​​​​​​O, EPAP +8 cm H_2_​​​​​​​O	Room air	Leadless pacemaker placed
Discharge (POD 7)	BCV −22/+8 cm H_2_O, RR 16 breaths/min, 1:1 sync	None	BCV continued for seven additional weeks
Two-month post-op	AVAPS resumed (original settings)	None	Return confirmed by follow-up titration PSG

**Figure 2:**
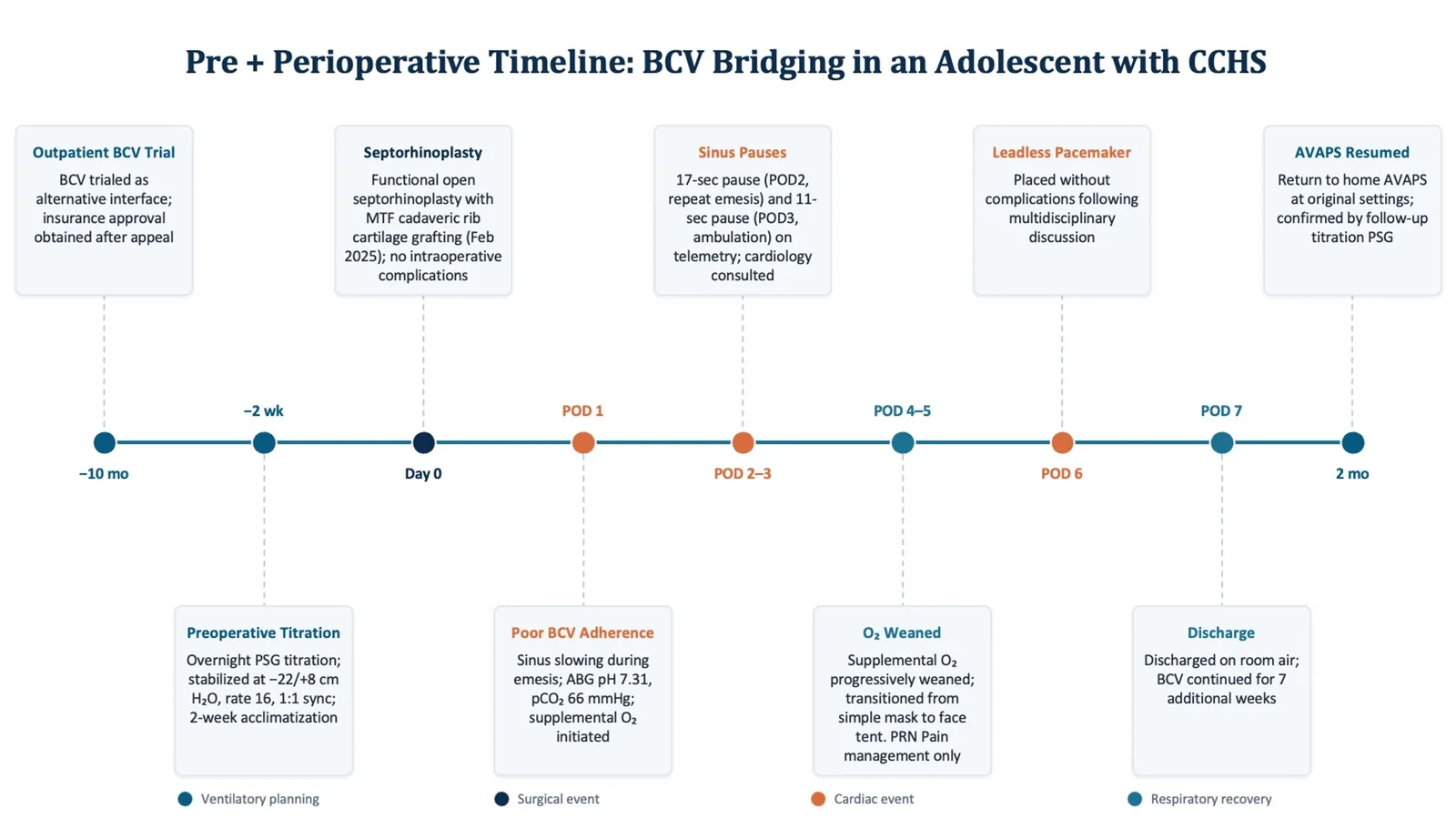
Pre + perioperative timeline: BCV bridging in an adolescent with CCHS. Pre- and perioperative timelines of BCV bridging in a patient with CCHS undergoing septorhinoplasty from preoperative device trial and titration through postoperative respiratory and cardiac events to eventual return to home AVAPS. BCV: biphasic cuirass ventilation; AVAPS: average volume-assured pressure support; POD: postoperative day; PSG: polysomnogram.

Supplemental oxygen requirements, initially elevated above the patient’s baseline, were weaned progressively and discontinued by POD 6 (Table 2). Outside of POD 1, BCV adherence was 7-8 hours per night. The patient was discharged on POD 7 without supplemental oxygen, continuing BCV for seven additional weeks. At the two-month follow-up, AVAPS was resumed at the original settings. Postoperative cardiology and otolaryngology follow-up were unremarkable. A full timeline of events is summarized in Figure 2.

## Discussion

This case demonstrates that BCV can provide adequate and well-tolerated nocturnal ventilatory support in a CCHS patient when positive pressure interfaces are temporarily contraindicated. Nasal surgery with cartilaginous grafting is an underappreciated situation in which standard NIV mask use becomes infeasible. To our knowledge, perioperative BCV use in this context has not been previously described in the CCHS literature. The preoperative home trial and titration polysomnography were important components, establishing that adequate ventilation was achievable on the device before the patient underwent surgery. Providers planning nasal surgery in CCHS patients should initiate this process well in advance of the planned procedure date.

As mentioned earlier, current guidelines for CCHS recognize four main ventilatory modalities: tracheostomy-based positive pressure ventilation, NIV positive pressure ventilation via mask interface, phrenic nerve pacing, and NPV [4]. All four modalities share a goal of correcting sleep-related hypoventilation, particularly during non-rapid eye movement (NREM) sleep, when CCHS patients exhibit their highest level of CO_2_ insensitivity [4]. Currently, mask-based NIV (including bilevel positive airway pressure (BiPAP) and AVAPS) is the preferred approach for older children and adults requiring nocturnal-only support, with transition from tracheostomy being pursued after 7-10 years of age [4,7]. NPV holds a unique advantage over its counterpart modalities, as it requires no nasal or facial interface, leaving the patient's face entirely free, an aspect critical to the current case [4].

The Hayek RTX BCV (Hayek Medical Devices, Philadelphia, Pennsylvania) device works by applying cyclical negative and positive extra-thoracic pressure via a fitted rigid chest shell, mimicking both phases of the respiratory cycle through a process closer to physiological breathing than does positive pressure ventilation [6,8]. Through the device's “control mode” (as used in our patient at -22/+8 cm H_2_O), the device delivers a mandatory backup rate independent of patient effort, which is well-suited to the absent respiratory drive present during NREM sleep in CCHS [6]. Recent comprehensive reviews of BCV for airway surgery further highlight its expanding role across ENT procedures, finding that BCV provides effective ventilation while preserving an unobstructed operative field and may be particularly valuable when conventional airway instrumentation is undesirable or infeasible [9]. Literature surrounding the usage of NPV in the setting of CCHS is limited but encouraging. DeRusso et al. reported nine children with CCHS supported by home NPV, seven of whom never required tracheostomy [6]. Furthermore, Kameyama et al. described a two-year-old with CCHS in whom BCV was used alongside NIV for two PODs following abdominal surgery to address potential atelectasis-related desaturation [10]. To our knowledge, this case may represent one of the earliest reported uses of BCV as a primary perioperative nocturnal ventilatory modality in CCHS, titrated and confirmed via overnight polysomnography before surgery.

The elevated and sustained oxygen requirements across the first five postoperative days warrant discussion. While poor device adherence on night 1 clearly contributed to the documented CO₂ retention, the persistence of supplemental oxygen need beyond that point (despite improving BCV adherence) suggests additional physiological factors. Postoperative nasal edema altering airway dynamics, residual anesthetic effects, and the physiological stress response in an autonomically compromised patient may all have played a role. Additional contributors to sustained oxygen requirements may have included postoperative atelectasis, transient ventilation-perfusion mismatch, reduced postoperative mobility, and pain-related shallow breathing. Perioperative analgesia consisted of scheduled/PRN acetaminophen and ketorlac; however, breakthrough pain on PODs 1 and 2 required PRN oxycodone (7.5 mg every four hours) and hydromorphone (0.25 mg every two hours), which may have contributed to a further depressed central ventilatory drive during this window. Pain was subsequently well-controlled with scheduled acetaminophen and ketorlac alone from POD 2 onward, a transition that coincided with the subsequent improvement in oxygen requirements. While difficult to pinpoint a single direct contributor, these mechanisms likely interacted with the patient's impaired autonomic ventilatory control to contribute to the temporary increase in oxygen requirements despite improving BCV adherence. This underscores that CCHS patients undergoing nasal surgery face a window of heightened ventilatory vulnerability that extends beyond device compliance alone and that close respiratory monitoring throughout the postoperative admission is warranted.

Perioperative management in CCHS also warrants further discussion, as CCHS patients require monitoring and ventilatory vigilance that extends throughout the entirety of each sleep period rather than simply the immediate postoperative recovery phase. Anesthetic agents further depress a central respiratory drive that is already diminished in CCHS, and opioids administered by any route can produce prolonged nocturnal hypoventilation in this population, as mentioned above within the circumstances of this case [11]. Thus, administration of opioid-sparing analgesics should be preferred when clinically feasible. Our patient's acute-on-chronic hypercapnic respiratory failure on POD 1 (pH 7.31, pCO_2_ 66 mmHg) in the setting of poor BCV adherence and breakthrough-pain-related opioid use illustrates that the first POD night may carry a greater ventilatory risk. Postoperative ICU or other high-acuity monitored care should be strongly considered for patients with CCHS requiring ventilatory support, undergoing major surgery, or in whom postoperative respiratory instability is anticipated. Current international CCHS guidelines emphasize continuous ventilatory monitoring through sleep and careful perioperative respiratory management, although they do not specifically mandate ICU admission for every surgical procedure [4]. This recommendation therefore reflects both published CCHS guidance and the experience of the present case.

The sinus pauses observed in this patient are consistent with previously documented cardiac risk in CCHS. Previous retrospective studies have found that 22% of CCHS patients demonstrated potentially life-threatening cardiac arrhythmias, with significant sinus pauses (three seconds) and/or cardiac symptoms across multiple PHOX2B genotypes, even those that are not considered high-risk [5]. Additionally, patients with PHOX2B mutations have impaired autonomic modulation that can lower the threshold for vagally mediated sinus node suppression, particularly during physiological stressors such as vomiting [5]. The perioperative setting, with nausea, pain, and altered autonomic tone, may have amplified these events in our patient. Clinicians managing CCHS patients perioperatively should maintain continuous telemetry and have a low threshold for cardiology consultation, even in patients without prior documented arrhythmias. Leadless pacemakers may represent a reasonable option in selected younger patients because they avoid transvenous leads and may reduce infection risk [12]; however, device selection should be individualized according to electrophysiologic findings, patient anatomy, anticipated pacing needs, and multidisciplinary assessment with experienced cardiologists.

Finally, the initial insurance denial for the BCV device, driven by the cosmetic component of the septorhinoplasty, posed a meaningful logistical barrier and should be anticipated when managing rare disease patients in elective surgical settings. Early and proactive engagement with payers is advisable. Limitations of this report include its single-patient design and lack of generalizability to other CCHS phenotypes. Further cases and prospective protocols are needed to guide perioperative ventilation management in CCHS patients undergoing nasal surgery.

## Conclusions

In summary, this case demonstrates that BCV can serve as a safe and effective perioperative ventilatory bridge in CCHS patients in settings in which mask-based interfaces are contraindicated, provided adequate preoperative titration is performed. The successful transition back to home AVAPS at two months confirms that temporary BCV use does not compromise long-term ventilatory management. Beyond the ventilatory findings, the occurrence of clinically significant sinus pauses in the perioperative setting reinforces the importance of continuous cardiac monitoring in all CCHS patients undergoing surgery, regardless of prior history. As the CCHS population ages and pursues elective surgical procedures, clinicians will increasingly encounter scenarios requiring individualized ventilatory planning. Although limited to a single patient, this report illustrates a practical multidisciplinary approach that may inform perioperative planning for selected CCHS patients in whom conventional NIV interfaces are temporarily contraindicated.
